# A Debate on Vitamin C: Supplementation on the Hotline for Critically Ill Patients with COVID-19

**DOI:** 10.34172/apb.2021.046

**Published:** 2020-08-17

**Authors:** Ata Mahmoodpoor, Ali Shamekh, Sarvin Sanaie

**Affiliations:** ^1^Anesthesiology and critical care department, Faculty of Medicine, Tabriz University of Medical Sciences, Tabriz, Iran.; ^2^Student Research Committee, Tabriz University of Medical Sciences, Tabriz, Iran.; ^3^Neurosciences Research Center, Aging Research Institute, Tabriz University of Medical Sciences, Tabriz, Iran.

## Dear Editor,

COVID-19, the newly emerged viral disease spreading with an unstoppable rate, has been in the center of attentions all over the world. The virus causes acute respiratory distress syndrome (ARDS) and has the most of its victims in more vulnerable groups such as the elderly and people with underlying diseases like diabetes or cardiopulmonary diseased.^1^ Same as any other newly emerged viral disease, there are no definite and curative treatments available for the COVID-19 till now, so almost all of the available therapies are designed to support the body against the virus. The body reacts to the environment according to its resources, and a healthy and complete nutrition is on the top of any supportive therapies against any kind of diseases and provides the resources to the body’s defense mechanisms. Body systems, defensive barriers and immune responses are all reinforced with the fuel provided by a complete and healthy nutrition, fighting the mighty COVID-19 is no exception. One of the most controversial nutrients of these days is vitamin C, claimed to have beneficial effects on COVID-19 by some authorities and being criticized by the others on the other hand. As it is known that sufficient vitamin C can reinforce the body’s first defense lines (i.e. the skin, mucus membranes, and other innate immune mechanisms) which can effectively protect against infectious diseases.^2^ Vitamin C deficiency has been shown to be related to the increased risk and severity of influenza infections. Vitamin C supplementation has shown promising results in the activation of the innate immune system against influenza A virus/ H1N1 infection, and was shown to reduce the replication cycle of the virus.There is evidence suggesting that the intake of vitamin C can also reduce the incidence of upper respiratory tract infection, especially among the female population.^3^ Low levels of vitamin C are common in critically ill patients due to decreased intake and increased needs. Because vitamin C is multifunctional, hypovitaminosis can increase the severity of illness and hinder the recovery. There are some case reports suggesting that high-dose vitamin C administration can be associated with rapid resolution of virus-induced ARDS without any final post-therapy fibrosis.^4^ Moreover, vitamin C has a physiologic role in sepsis including attenuation of oxidative stress and inflammation, improvement in vasopressor synthesis and endovascular function, enhancement of immune cell function, down-regulation of inflammatory genes, and epigenetic immunologic modifications. During the cytokine storm following infection, accumulation of neutrophils in the lung destroys alveolar capillaries which can be prevented by vitamin C. Moreover, vitamin C reduces alveolar epithelial water channel damage which leads to clearance of alveolar fluid. Meanwhile, vitamin C can prevent the formation of neutrophil extracellular traps, a biological event of vascular injury caused by neutrophil activation.^5^ Marik suggested that a combination of Hydrocortisone, Ascorbic Acid and Thiamine (HAT therapy) can reduce organ failure and mortality in septic patients. These components, one of which is vitamin C, can act synergistically and restore the dysregulated immune response.^6^ The CITRIS-ALI study found that vitamin C supplementation could not modify C reactive protein, thrombomodulin, and Sequential Organ Failure Assessment (SOFA) Score. However, the mortality rate was significantly reduced by 16.5%, ICU and hospital length of stay by 3.2 and 6.7 days, respectively, and mechanical ventilation requirements by 2.5 days. It has been suggested that ineffectiveness of vitamin C on biomarkers can be due to survivorship bias; i.e. the samples were taken at 96 hours, time when more severely ill patients were excluded from the study because they had died.^7^ A meta-analyses that included five studies with 142 critically ill patients concluded that intravenous vitamin C administration showed no adverse reactions, and reduced duration of mechanical ventilation and need for vasopressor support, but could not show any effect on mortality.^8^ Another meta-analysis has evaluated the effects of various doses of vitamin C on the mortality of 1210 critically ill adults. Intravenous vitamin C in doses of 3-10 g/d reduces the overall mortality rates (OR: 0.25; 95% CI: 0.14–0.46; *P* < 0.001; *I*^2^ = 0.0%), but doses less than 3 g/d or more than 10 g/d had no effect. Moreover, intravenous vitamin C resulted in reduced duration of mechanical ventilation and vasopressor support.^9^

Regarding the evidence around vitamin C’s effect on immune function, it has been shown to increase differentiation and proliferation of B- and T-lymphocytes due its gene regulating effects. As vitamin C deficiency results in impaired immunity and higher susceptibility to infection, prophylactic prevention of infection requires dietary vitamin C intakes to optimize its cellular and tissue levels. On the other hand, treatment of proven infection requires significantly higher doses of vitamin C to optimize immune dysfunction and hyper-inflammatory response and metabolic demand.^10^

Although mega-doses of oral vitamin C does not seem to be protective against coronavirus, trials are currently underway testing the ability of intravenous vitamin C to protect against the effect of a cytokine storm. There is conflicting information about the effect of vitamin C in COVID-19 considering heterogeneity of the preliminary studies.Therefore, during the current pandemic of COVID-19, it is necessary to study the clinical efficacy and safety of vitamin C for viral pneumonia through randomized controlled trials. At the moment, there are more than 10 clinical trials running in the world evaluating the effects of vitamin C in patients with COVID-19 infection. In these trials, various doses of intravenous or oral vitamin C are being tested and we have to wait at least till October, 2020 to see the results. Till achieving high level of evidence and considering all abovementioned data and mechanisms, it seems that vitamin C supplementation may be beneficial to any respiratory viral infection, including COVID-19 because of its low cost and safety profile. However, physicians should consider the followings when administering vitamin C to patients with ARDS due to COVID-19: precision medicine, patients’ characteristics, organ dysfunctions especially kidney injury, severity scores, vitamin C dose (3-10 g/d), rout of administration (oral versus intravenous), and method of administration (bolus versus continuous infusion).

## Ethical Issues

Not applicable.

## Conflict of Interest

The authors have no conflict of interest to report.
